# Prevalence of malocclusion and assessment of orthodontic treatment needs among Syrian refugee children and adolescents: a cross-sectional study

**DOI:** 10.1186/s12903-021-01663-4

**Published:** 2021-06-14

**Authors:** Nesreen A. Salim, Mariam M. Al-Abdullah, Abeer S. AlHamdan, Julian D. Satterthwaite

**Affiliations:** 1 Prosthodontic Department, Faculty of Dentistry, The University of Jordan, Amman, 11942 Jordan; 2 The University of Jordan Hospital, Amman, Jordan; 3 Department of Paediatric Dentistry, Orthodontics, and Preventive Dentistry, Faculty of Dentistry, The University of Jordan, Amman, 11942 Jordan; 4 The University of Jordan, Amman, Jordan; 5 Restorative Dentistry, Division of Dentistry, School of Medical Sciences, University of Manchester, Oxford Road, Manchester, M13 9PL UK

**Keywords:** Malocclusion, IOTN/DHC, Refugee, Orthodontic treatment need

## Abstract

**Background:**

There is a scarcity of data concerning the prevalence and pattern of malocclusion and orthodontic treatment needs in Syrian refugee. In this study, extra and intra-oral features of malocclusion and the dental health component of the Index of Orthodontic Treatment Need (IOTN) were reported.

**Methods:**

Examination of 606 Syrian children/adolescents refugees attending Zaatari clinic was carried out (males = 280, females = 326, mean age = 11.84 ± 2.1 years). Subjects not within the age limit, with a history of orthodontic treatment, or with craniofacial anomalies were excluded. Both extra and intra-oral features of malocclusion were assessed. Intra-oral features included inter- and intra-arch occlusal characteristics: crowding, spacing, crossbite, overjet, overbite, molar and canine relationship, incisor relationship, and centerline shift. In addition, the dental health component (DHC) of the Index of Orthodontic Treatment Need (IOTN) was recorded. Gender and age variations in malocclusion characteristics and IOTN grading were tested using chi-square and nonparametric tests respectively (*P* < 0.05).

**Results:**

The prevalence of malocclusion was 83.8% (52.6% class I, 24.2% class II, 7% class III). The most common features of malocclusion were crowding (71.1%) followed by centerline shift (52.1%), increased overjet (36.1%), high vertical proportions (34%) and deep overbite (31.2%); there were significant gender and age differences for a number of occlusal traits. The prevalence of moderate to severe need for orthodontic treatment was 67.7%.

**Conclusions:**

This study provides baseline data on the prevalence of malocclusion in Syrian refugee children/adolescents in Zaatari camp where data concerning oral health of this population are lacking. The prevalence of orthodontic treatment need was high warranting the need for a comprehensive interceptive orthodontic program to prevent increasing oral health problems in the future. This high burden of oral diseases has a negative financial impact on the hosting country which can be reduced through public health interventions and implementing community-based dental healthcare for this underprivileged population.

## Background

The Syrian conflict, which began in March 2011, has forced an estimated 6.6 million Syrians to flee their country and another 6 million people have been displaced inside the country [1]. As the conflict continues, with new arrivals in host countries this population is expected to increase [1]. Jordan hosts more than 657,628 Syrian refugees, with 76,989 refugees residing in Zaatari camp, of which 55.9% are younger than 18 years. Zaatari refugee camp is the largest camp in Jordan and the second largest camp worldwide [1].

Refugees have limited access to healthcare services and host communities struggle to meet health needs (including dental care) of growing refugee populations. Assessment of oral health status, unmet dental needs and accessibility to dental services is paramount to target urgent needs efficiently [2, 3].

Refugee children are particularly vulnerable to oral diseases [4]. Malocclusion is a common oral health problem, after tooth decay and periodontal disease, and is ranked third in dental public health priorities worldwide [5, 6]. Malocclusions have been associated with psychosocial distress, discomfort, low quality of life, poor periodontal condition, and impaired masticatory function. Assessing malocclusion in childhood may minimize or eliminate future treatment needs, reduce treatment cost and help plan preventative and curative measures [7] and reporting orthodontic treatment needs is important for resource planning and funding [8, 9].

Prevalence of malocclusion has been studied in different populations [5, 6, 10–18]. However, no studies have been done relating to refugees in general and Zaatari camp refugees in particular. The aim of this study was to investigate the prevalence of malocclusion and orthodontic treatment needs in Syrian refugee children/adolescents; the objectives were to:

1. assess the prevalence of malocclusion and associated occlusal traits
2. ascertain the orthodontic treatment need using Dental Health Component (DHC) of Index for Orthodontic Treatment Need (IOTN)

## Methods

### Ethical approval

The research protocol was approved by the Research Ethical Committee of the School of Dentistry of the University of Jordan (75/2019/71) and in full accordance with the world medical declaration of Helsinki. For each visit authorization was obtained to enter the camp and written informed consent was obtained from parents. The study is reported in line with strengthening the Reporting of Observational Studies in Epidemiology (STROBE) recommendations for cross-sectional studies [19].

### Study design

A prospective cross-sectional clinical survey was conducted from July to September 2019 to assess malocclusion and associated occlusal features, and to record the DHC/IOTN among Syrian refugee children/adolescents aged 7–19 years residing in Zaatari camp (Table 1). Subjects not within the age limit, with a history of orthodontic treatment, or with craniofacial anomalies were excluded. 606 individuals were examined in Zaatari dental clinics by two examiners; an orthodontist (M A) and a prosthodontist (N SA), assisted by four junior dentists. The clinical examination and data collection was performed in dental units available in Zaatari camp, under artificial illumination using a plane mouth mirror and a millimeter ruler (x-ray facilities were not available). Examination and data collection were conducted during school holidays to ensure the sample required in terms of numbers and age range was recruited. The clinics had a high level of attendance, and there was thus no need for further active recruitment.

**Table 1:** Data collected in the study

Demographic and general dental data	Gender, age, history of dental trauma, and oral habits
Data on malocclusion *Extra-oral features:* Patients were seated in the natural head position and the following were recorded	1. Facial profile relationship in the antero-posterior dimension (Class I, II, and III): the deepest concavity of the anterior surface of the maxilla (soft tissue A point) relative to the deepest concavity of the anterior surface of the mandible (soft tissue B point) within the sagittal plane 2. Facial profile convexity: assessed using an imaginary line connecting the glabella, subnasale, and pogonion 3. Vertical proportions of the face assessed by soft tissue Frankfort-mandibular plane angle (FMPA): The angle between Frankfort plane and the line representing the lower border of the mandible was examined relative to the occipital area. This was classified into high, average or low (both lines meet anterior to the occipital, at the occipital, posterior to the occipital; respectively) 4. Nasolabial angle (NLA): classified into acute (< 90 degrees), average (90–110 degrees), or obtuse (> 110 degrees) 5. Lips at rest: competent (meet at rest), incompetent (separated > 2 mm at rest) 6. Upper and lower lips were described as prominent (everted), average, or retrusive (inverted) relative to the true vertical line extending from subnasale
*Intra-oral features*	1. Crowding in both arches: no crowding, mild (2–4 mm), moderate (4–8 mm), or severe (> 8 mm) 2. Spacing in both arches: no spacing, localized, or generalized 3. Contact point deflection: no displacement, < 1 mm, 1–2 mm, 2–4 mm, or > 4 mm 4. Centerlines (CL): Upper/lower dental centerlines were examined relative to the face centreline and recorded as centred, or shifted 5. The right/left molar and canine relationship was recorded to the nearest full unit and was classified into Class I, II, or III. If the first permanent molars and/or canines were missing, no registration was made 6. Crossbite: a transverse discrepancy in the buccal segment affecting two or more teeth (no crossbite, buccal, or lingual crossbite (scissor bite)) 7. Mandibular displacement (no, anterior or lateral displacement) in the presence of anterior or posterior crossbite 8. Discrepancy of the position of the head of the condyle between retrusive contact position (RCP) and intercuspal position (ICP) was examined (no discrepancy or a discrepancy of < 1 mm, 1–2 mm, or > 2 mm) 9. Incisor relationship: classified in the maximum intercuspation as Class I, Class II division 1, Class II division 2, or Class III 10. Overjet (OJ): recorded as average (2–4 mm), increased (> 4 mm), reduced (0–2 mm), or reversed (on at least two incisors) 11. Overbite (OB): recorded as increased, average, decreased, or anterior open bite (AOB)
Orthodontic need	The DHC of the IOTN: grade 1 (no need), grade 2 (little need), grade 3 (moderate need), grade 4 (great need), or grade 5 (very great need)

Sample size was calculated prior to data collection using expected sample size formula depending on previous studies in other countries [5, 6]. Since the reported prevalence showed wide variation, we used an expected ratio of 50% which produce the maximum needed sample [20]. Our target population were children and adolescents in Zaatari camp (around 37,000) and with a power of 85%, alpha value of 0.05 (a margin of error of 5%), and a confidence interval of 95% the calculated sample was 381 [21]. Therefore we aimed for a sample of around 600 to take into consideration that our study included multiple comparisons of proportions and ranked values.

### Examiner calibration

In order to reduce bias in data collection, prior to the assessments, examiners underwent training on basic orthodontic examination. Subsequently, 12 sample cases (that covered all orthodontic features to be examined) were assessed by each examiner (the exercise being repeated after 4 weeks). Inter-examiner correlation coefficient was 0.94 initially and 0.91 after 4 weeks, indicating very good reliability.

### Examination procedure

**Fig. 1:**
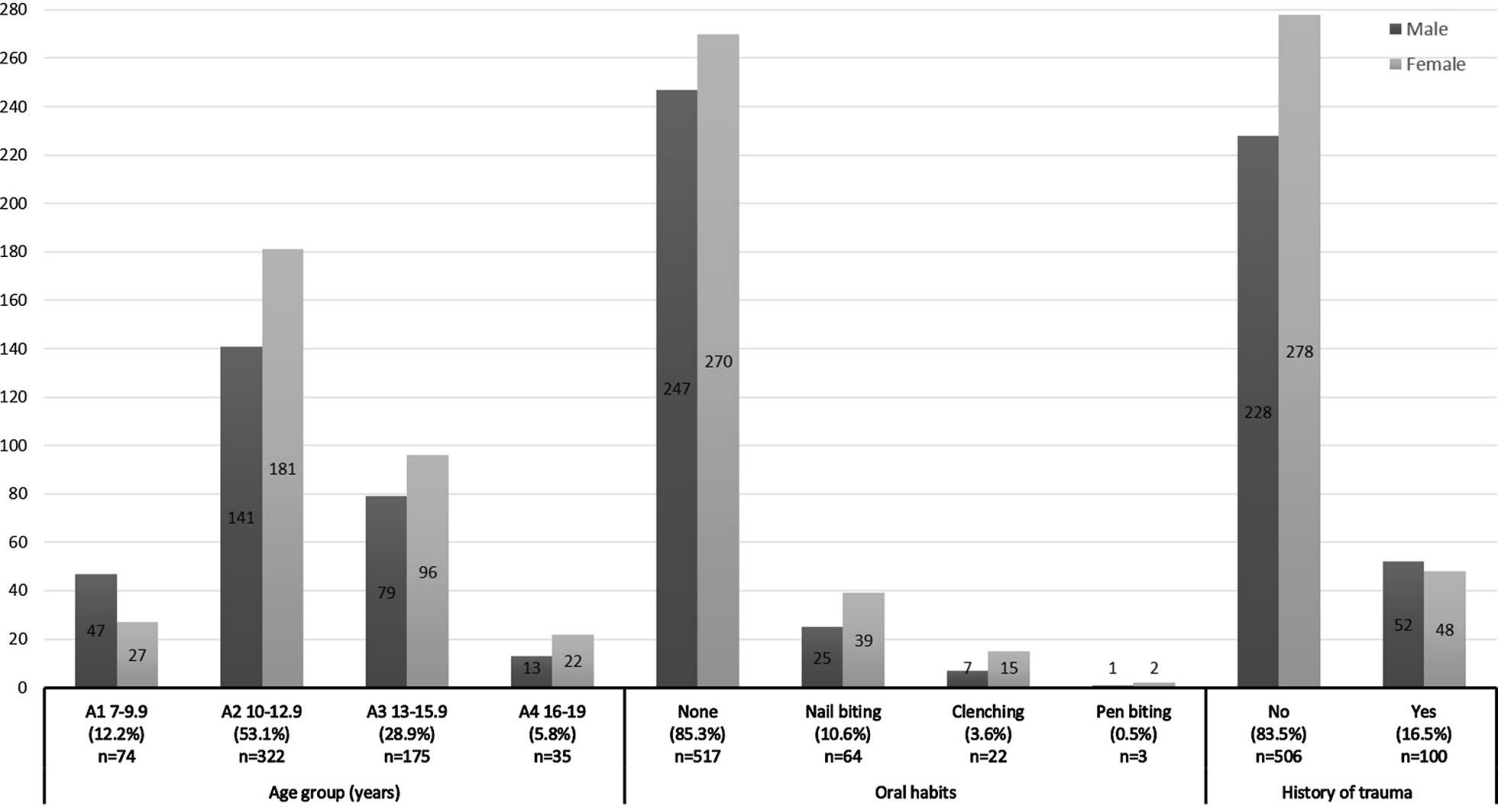
Sociodemographic characteristics and oral habits of the study sample (N = 606). (Age groups: **A1** (early mixed dentition): 7–9.9 years., **A2** (late mixed dentition): 10–12.9 years., **A3** (early permanent dentition): 13–15.9 years., and **A4** (permanent dentition): 16–19 years)

Gender, age, history of dental trauma, and oral habits were recorded (Fig. 1). Age was categorized into: A1 (early mixed dentition): 7–9.9 years., A2 (late mixed dentition): 10–12.9 years., A3 (early permanent dentition): 13–15.9 years., and A4 (permanent dentition): 16–19 yrs. In addition to oral habits which included nail biting, clenching, pen biting and thumb sucking.

For each participant a comprehensive orthodontic examination was undertaken, with a proforma being used to collect data as outlined in Table 1. The main malocclusion trait used for general classification was the basic Angle’s classification [22]. In cases of missing first permanent molar(s), early loss of primary molar(s), and/or asymmetric molar relationship, the predominant pattern of the occlusion was used. Each subject was then classified to one of four groups: Class I normal occlusion (where the deviation of the ideal occlusion was mild and would not compromise dental aesthetic or function), Class I malocclusion, Class II malocclusion, and Class III malocclusion.

### Statistical analysis

SPSS version 25.0 (Armonk, NY: IBM Corp., 2017) was used for statistical analysis. Descriptive analysis and frequency tables were used for general description of the results. Chi-square and binomial tests of significance were performed to identify significant differences in the prevalence and pattern of malocclusion between gender and age groups. The ranking of the IOTN was compared between genders using Wilcoxon-Mann-Whiney (U) test and between age groups using independent sample Kruskal–Wallis test (*P* < 0.05 significance level).

## Results

### Demographic data

All eligible participants during the study period were invited to take part in the study and were subsequently included: none declined or were excluded. Demographic data is shown in Fig. 1. Of 606 patients, 280 (46.2%) were male and 326 (53.8%) were female, with age range 7 to 19 years (mean = 11.84, SD = 2.1); the majority (53.1%) were in A2 age group (10–12.9 years). Regarding oral habits, none reported thumb sucking. 16.5% had a history of dental trauma with males (19.0%) affected more than females (15.0%) but not to a significant level (*P* = 0.20).

### Malocclusion classifications

16.2% had normal occlusion, 52.6% had class I, 24.2% had class II, and 7% had class III malocclusion.

### Extra-oral features

Extra-oral features are shown in Table 2. The antero-posterior pattern of the facial profile was Class I in 59.2% of the sample, Class II in 30% of the sample and Class III in 10.7% of the sample. 48.2% had a convex profile and 4% had a concave profile. 50.7% of patients had an average FMPA. NLA was average in 75.2%. Most had competent lips (72.6%). Average prominence of the upper and lower lips was common (64.7%, 77.9% respectively), while a retrusive upper lip was more common (21.6%) than lower lip (2.0%), but a prominent lower lip was more common (20.1%) than the upper lip (13.7%).

**Table 2:** The distribution of extra-oral features of malocclusion according to gender

Parameters		Gender N (%)		X^2^ (*P*)	Total (N = 606)	
		Male (N = 280)	Female (N = 326)		N	%
Antero-posterior facial profile pattern	Class I	172 (61.0)	187 (57.4)	1.59 (0.45)	359	59.2
	Class II	77 (28.0)	105 (32.2)		182	30.0
	Class III	31 (11.0)	34 (10.4)		65	10.7
Facial convexity	Convex	134 (48.0)	158 (48.0)	1.83 (0.40)	292	48.2
	Straight	138 (49.0)	152 (47.0)		290	47.9
	Concave	8 (3.00)	16 (5.00)		24	4.00
Soft tissue FMPA	High	93 (33.0)	113 (35.0)	6.38 (0.041)*	206	34.0
	Average	133 (48.0)	174 (53.0)		307	50.7
	Low*	54 (19.0)	39 (12.0)		93	15.3
NLA	Acute	37 (13.0)	47 (14.0)	1.15 (0.56)	84	13.9
	Average	216 (77.0)	240 (74.0)		456	75.2
	Obtuse	27 (10.0)	39 (12.0)		66	10.9
Lip competency	Competent	202 (72.0)	238 (73.0)	0.056 (0.81)	440	72.6
	Incompetent	78 (28.0)	88 (27.0)		166	27.4
Upper lip prominence	Prominent	40 (14.3)	43 (13.0)	0.153 (0.93)	83	13.7
	Average	180 (64.3)	212 (65.0)		392	64.7
	Retrusive	60 (21.4)	71 (22.0)		131	21.6
Lower lip prominence	Prominent	59 (21.0)	63 (19.3)	2.47 (0.29)	122	20.1
	Average	213 (76.0)	259 (79.5)		472	77.9
	Retrusive	8 (3.00)	4 (1.20)		12	2.00

*N* number of subjects, *FMPA* Frankfort mandibular plane angle, *NLA* Nasolabial angle

^*^Significance difference between males and females at *P* < 0.05

There was no significant difference in the gender distribution of extra-oral features of malocclusion except for the soft tissue FMPA, where those with a low FMPA (15.3%) were mostly males (*P* = 0.014). In relation to age there were no significant differences except for the upper lip prominency (*P* = 0.009) which was less prevalent in age group A2 (9.0%, *P* = 0.045) compared to other age groups (A1 = 18.9%, A3 = 19.4%, A4 = 17.1%). Competency of lips was the only extra-oral feature significantly associated with dental trauma, where 22.9% of patients with incompetent lips experienced dental trauma compared to 14.1% with competent lips (*P* = 0.009).

### Intra-oral features

#### Intra-arch characteristics

Intra-arch characteristics are shown in Table 3. 71.1% of the sample had crowding with 47.2% having crowding in both arches, 9.6% in upper arch only, and 14.4% in lower arch only. Females showed more crowding than males with a significant association between upper arch crowding and gender (*P* < 0.001); females with mild crowding in the upper arch was significantly more than males (*P* = 0.026). In the lower arch there was no significant difference in crowding between genders. Females had more severe deflection of contact points (> 4 mm) between teeth in both upper and lower arches, but this was not significant when compared to males.

**Table 3:** Intra-oral features (intra-arch characteristics) of malocclusion within gender groups

Parameters		Upper arch				Lower arch			
		Gender N (%)			Total (N = 606)	Gender N (%)			Total (N = 606)
		Male N = 280	Female N = 326	X^2^ (*P*)	N (%)	Male N = 280	Female N = 326	X^2^ (*P*)	N (%)
Crowding	None	140* (50.0)	123* (38.0)	10.12 (0.018)*	263 (43.4)	107 (38.0)	125 (38.3)	1.10 (0.78)	232 (38.3)
	Mild < 4 mm	59* (21.0)	96* (29.0)		155 (25.6)	103 (37.0)	109 (33.4)		212 (35.0)
	Moderate 4–8 mm	41 (15.0)	53 (16.0)		94 (15.5)	37 (13.0)	50 (15.3)		87 (14.4)
	Severe > 8 mm	40 (14.0)	54 (17.0)		94 (15.5)	33 (12.0)	42 (13.0)		75 (12.4)
Deflection of contact points	None	75 (27.0)	74 (23.0)	6.73 (0.15)	149 (24.6)	58 (21.0)	69 (21.0)	5.17 (0.27)	127 (21.0)
	< 1 mm	47 (17.0)	37 (11.0)		84 (13.9)	74 (26.0)	76 (23.3)		150 (24.8)
	1–2 mm	56 (20.0)	73 (22.0)		129 (21.3)	59 (21.0)	76 (23.3)		135 (22.3)
	2–4 mm	51 (18.0)	67 (21.0)		118 (19.5)	53 (19.0)	47 (14.4)		100 (16.5)
	> 4 mm	51 (18.0)	75 (23.0)		126 (20.8)	36 (13.0)	58 (18.0)		94 (15.5)
Spacing	None	185* (66.0)	247* (76.0)	6.92 (0.031)*	432 (71.3)	227 (81.0)	267 (82.0)	2.23 (0.33)	494 (81.5)
	Localized	52 (19.0)	43 (13.0)		95 (15.7)	25 (9.00)	36 (11.0)		61 (10.1)
	Generalized	43 (15.0)	36 (11.0)		79 (13.0)	28 (10.0)	23 (7.00)		51 (8.40)
Centerline (CL)	Centered	238 (85.0)	260 (80.0)	2.83 (0.093)	498 (82.2)	166 (59.0)	191(59.0)	0.03 (0.86)	357 (58.9)
	CL shift	42 (15.0)	66 (20.0)		108 (17.8)	114 (41.0)	135 (41.0)		249 (41.1)

*U* upper arch, *L* lower arch, *N* number of subjects, Chi-square test

^*^Significance difference between males and females at *P* < 0.05

33.8% of the sample had spacing with 13.4% in both arches, 15.3% upper arch only, and 5.1% lower arch only. Chi-square test disclosed significant association between upper arch spacing and gender (*P* = 0.031), although further analysis between groups showed no significant difference between genders (P˃0.05). Lower arch spacing was less common than in the upper arch with no significant difference between genders.

Upper arch spacing was significantly different between age groups (*P* = 0.042); localized spacing in A1 age group (28.4%) was significantly higher (*P* = 0.016) than A4 group (5.7%).

The upper centerline was shifted (17.8%) less frequently than the lower centerline (41.1%). Upper centerline shift and/or lower centerline shift was seen in 52.1% of the cases, with no significant difference according to gender or age.

#### Inter-arch characteristics

Inter-arch characteristics are shown in Table 4. The majority showed class I molar relationship (54.8%), 15.0% had Class II and 3.8% had class III (asymmetric molar relationship was 24.8%). Similarly, the most common canine relationship was class I (32.2%), followed by class II (17.5) then class III (1.7%) (asymmetrical canine relationship was 20.3%). Distribution of molar and canine relationships between sides and genders was not significantly different.

**Table 4:** Intra-oral features (Inter-arch characteristics) of malocclusion within gender groups

Parameters		Gender N (%)				Total (N = 606)			
						R		L	
		Male (N = 280)		Female (N = 326)		N	%	N	%
		R	L	R	L				
Molar relationship	2° first molars not present	8 (3.00)	9 (3.00)	15 (5.00)	14 (4.00)	23	3.80	23	3.80
	Class I	188 (67.0)	192 (69.0)	210 (64.0)	198 (61.0)	398	65.7	390	64.4
	Class II	68 (24.0)	62 (22.0)	78 (24.0)	85 (26.0)	146	24.1	147	24.3
	Class III	16 (6.00)	17 (6.00)	23 (7.00)	29 (9.00)	39	6.4	46	7.6
Canine relationship	2° canines are not present	91 (32.5)	92 (33.0)	98 (30)	103 (31.5)	189	31.2	195	32.2
	Class I	115 (41.0)	119 (42.0)	132 (40.5)	119 (36.5)	247	40.8	238	39.3
	Class II	64 (23.0)	64 (23.0)	85 (26.0)	90 (28.00)	149	24.6	154	25.4
	Class III	10 (4.50)	5 (2.00)	11 (3.50)	14 (4.00)	21	3.5	19	3.1
Crossbite	None	223 (79.6)		259 (79.0)		482		79.5%	
	Buccal crossbite	52 (18.6)		65 (20.0)		117		19.3%	
	Lingual crossbite	5 (1.80)		2 (1.00)		7		1.20%	
Mandibular displacement (MD)	None	264 (94)		313 (96.0)		577		95.2%	
	Anterior MD	8 (3)		6 (2.00)		14		2.30%	
	Lateral MD	8 (3)		7 (2.00)		15		2.50%	
Discrepancy between the RCP and ICP	None	254 (91.0)		287 (88.0)		541		89.3%	
	< 1 mm	12 (4.00)		19 (6.0)		31		5.10%	
	1–2 mm	11 (4.00)		16 (5.0)		27		4.50%	
	> 2 mm	3 (1.00)		4 (1.0)		7		1.20%	
Incisors relationship	Class I	114 (41.0)		131 (40.0)		245		40.4%	
	Class II d1	70 (25.0)		80 (25.0)		150		24.8%	
	Class II d 2	56 (20.0)		56 (17.0)		112		18.5%	
	Class III	40 (14.0)		59 (18.0)		99		16.3%	
Overjet	Average	132 (47.0)		147 (45.0)		279		46.0%	
	Increased	101 (36.0)		118 (36.0)		219		36.1%	
	Reduced	39 (14.0)		54 (17.0)		93		15.3%	
	Reversed	8 (3.00)		7 (2.00)		15		2.50%	
Overbite	Average	93 (33.2)		109 (33.4)		202		33.3%	
	Increased	98 (35.0)		91 (28.0)		189		31.2%	
	Reduced	74 (26.4)		105 (32.2)		179		29.5%	
	AOB	15 (5.40)		21 (6.40)		36		5.90%	

*R* right side, *L* left side, 2°: permanent; *N* number of subjects, *d 1* division I, *d 2* division II, *AOB* anterior open bite

Crossbite, mandibular displacement, RCP-ICP discrepancy were equally distributed between genders. Posterior crossbite was present in 20.4% of patients, of which 94.4% had buccal and 5.6% had lingual crossbite.

Of the total, 40.4% had class I incisor relationship, 24.8% had Class II division 1 and 18.5% class II division 2: least common was class III (16.3%). 46.0% of the sample had an average overjet, and 33.3% had an average overbite. AOB prevalence was 5.9%, with A1 (12.2%) and A4 (14.3%) age groups more significantly affected than A2 (4.7%) and A3 (4.0%) groups (*P* = 0.012). All comparisons of occlusal relationships of the anterior segment between genders were insignificant *P* > 0.05.

#### DHC of the IOTN

DHC/IOTN data are shown in Table 5. Grade 4 was the most prevalent grade (34.8%). Grades 1 and 2, representing no/little need for orthodontic treatment was 32.3%, while grade 3, 4 and 5 (moderate/great need for orthodontic treatment) was 67.7%. No significant difference was detected according to gender or age in orthodontic treatment need (P˃0.05).

**Table 5:** Prevalence and distribution of the IOTN of the total sample and within gender

Index on orthodontic treatment need (DHC)		I-Gender N (%)		
		Males N = 280 (%)	Females N = 326 (%)	Total N (%)
*Grades and treatment needs*				
Grade 1	No need	53 (19.0)	49 (15.0)	102 (16.83)
Grade 2	Little need	41 (15.0)	53 (16.0)	94 (15.51)
Grade 3	Borderline need	81 (29.0)	82 (25.0)	163 (26.90)
Grade 4	Definitive need	88 (31.0)	123 (38.0)	211 (34.82)
Grade 5		17 (6.00)	19 (6.00)	36 (5.94)

## Discussion

The current research investigated the prevalence of malocclusion and orthodontic needs among Syrian children/adolescents in a large refugee camp in Jordan for the first time. Understanding the pattern of malocclusion and its severity can help in evaluating the dental orthodontic treatment needs and the oral health status of the studied population. In addition, findings of the present study will aid to design a tailored oral health promotion and treatment programs for Syrian children living in refugee camps with limited resources.

Refugees are more susceptible to a variety of illnesses, including dental problems [23]. Latest reports have shown that refugees have a higher incidence of dental caries and impaired oral hygiene than host people [2–4]. Untreated dental diseases can result in tooth decay/loss, which can lead to unhealthy eating habits and a decline in quality of life [23]. A previous study found that the most frequent treatment given to refugee children was extraction, which indicates poor oral hygiene and the refugees' propensity to seek dental services late in the course of their illness, mostly for emergency treatment [4, 24]. This finding, on the other hand, may suggest a shortage of restorative treatment services due to insufficient funding and delayed access to dental care [2–4, 24]. These extraction findings contrast negatively with those of Jordanian children who attended private and public schools, where only 19% of those children had missing teeth. These disparities can be due to socio-economic conditions and dental care accessibility [25].

Early primary tooth loss, on the other hand, causes occlusal disturbances and space loss in children, which may make subsequent dental care more difficult [25, 26]. Tooth loss was shown to be closely linked to the number of dental appointments, indicating a deterioration in oral health and likely a lack of interest in more conservative care choices [4]. Furthermore, a common misconception among parents is that primary teeth do not need attention because new teeth can erupt eventually; this indicates a lack of parental knowledge and poor attitudes toward primary dental care [4, 7]. Additionally, behavioral and habitual causes such as dietary habits, a shortage of dental care and prevention measures, and inadequate oral health can all be attributed to the high incidence of dental caries. Furthermore, it has been shown that a significant number of extractions were performed at the behest of parents, including the fact that the tooth might have been healed, but the patient/parent insisted on extraction [4]. This is due to a lack of knowledge and negative views about the value of dental care of primary teeth [2–4, 24]. Furthermore, except in cases of pain-related emergencies, dental treatment is not commonly considered a primary concern for refugees, as access to dental facilities is a significant obstacle. [3, 4, 24].

### Malocclusion classification

The reported prevalence of normal occlusion ranges widely (e.g., from 6.5% in Latino adolescents [27] to 67.3% in British school children [28]). This might explained by variation in registration methods, indices used, era of the research, ethnic origin, and dental development stage [22]. A recent study, which compared dentofacial differences between Syrian and European adolescents with Class II division I malocclusion, highlighted that ethnic differences are important considerations for orthodontic diagnosis and treatment [29].

Most studies have reported that class I malocclusion is most common followed by Class II and class III [7, 12, 15, 16, 27]; although the percentages found in our study differed from previous studies, the pattern was similar.

### Extra-oral features

The prevalence of the antero-posterior pattern of the facial profile in the current study followed closely the prevalence of the general classification of the malocclusion. This could be explained by the use of Angle’s classification which is based on the first permanent molar position in the jaw and in the absence of local dental factors such as early loss of primary teeth, the molar relationship will be directly affected by the antero-posterior position of the jaws and thereafter the Angle’s classifications. Similarly, patients with Class I and II malocclusions usually present with straight to convex profile, hence only a small proportion (4%) had a concave profile which is closely related to the prevalence of Class III malocclusion in this study: this is in agreement with a previous study [13].

For the rest of the extra-oral features, the majority of our sample presented with average values. However, the percentage of males who had low FMPA was significantly higher than females (related to an anterior growth rotation tendency and possible reduction in the anterior lower facial height), although previous studies have reported no difference in FMPA between genders [30, 31].

Patients with incompetent lips had significantly higher prevalence of dental trauma than patients with competent lips, and this matches existing data that improper lip coverage increases the risk of dental trauma 2.18 times [32]. Previous reports of prevalence of dental trauma are comparable to our results, with- incompetent lips being one of the significant risk factors with males affected more than females [33].

### Intra-oral features

#### Intra-arch characteristics

Crowding was common in this study, (71.1%), comparable to those reported for an Iranian study [12], but greater than those reported by studies with compatible pattern of crowding [10–13, 17]. Lower prevalence of crowding has been reported in Nigerians (20.1%) [16] and Tanzanians (14.1%) [6], explained by the ethnic origin of the sample where having a spaced primary dentition and large arches in black people was a common finding.

Most studies investigating crowding show no significant difference between gender [6, 11, 12, 16, 17] and this is in agreement with our results for the lower arch only. The high prevalence of crowding in our population could be attributed to the poor/limited dental services that might lead to loss of contact points due to proximal caries [4], or early loss of deciduous teeth without access to interceptive space maintainers.

The prevalence of spacing in one/both arches is consistent with previous findings; for example, in Saudians (27.2%) [13], in Jordanians (26.7%) [10] and in Colombians as (25.9%) [17], although lower rates are reported for Tanzanians [6], Chinese and Iranian adolescents [12, 15]. Similar to our study, some investigations [6, 12, 17] have shown that males have a higher prevalence of spacing than females.

Few studies have reported centerline shift, with data only for ˃2 mm [6, 12, 17]. This could explain why the prevalence of centerline shift in our study (52.1%) was higher than previous studies (31.7% [10], 23.7% [12], 22.5% [6] and 13.2% [17]) where we recorded even mild centerline shift (< 2 mm). Similar results have been reported (53.8%) [18].

#### Inter-arch characteristics

Unlike our study, most studies report molar relationship combining both sides [10, 12], without recording asymmetric findings [6, 13, 17] or describe molar and canine relationship as one entity [14]. Nevertheless, a common finding is a similar pattern with the most common molar relationship recorded as class I followed by class II and finally class III. The prevalence of asymmetric molar relationship and canine relationship in our study was higher than that reported for Jordanian children (17.7%) [10] and Iranian adolescents (18.3%) [12]. The most common aetiology for asymmetric molar relationship is early loss of deciduous molars [24]. This again highlights that that our sample population had limited access to dental services, poor management of carious deciduous teeth, with extraction being the most provided treatment without interceptive measures to prevent space loss, where primary lower molars were the most commonly extracted teeth [4].

The prevalence of buccal crossbite in our study was greater than that reported in previous studies [5, 10–15, 17, 18], although our study and most previous studies, have reported that the buccal crossbite has higher prevalence than lingual. Some previous studies consider anterior crossbite even only one anterior tooth was affected [6, 11, 12, 14]., and thus report a higher prevalence than our results (which considered at least two incisors) However, in Jordanian school children the anterior crossbite has been reported as, lower than our results, which could be explained by the comparatively higher percentage of class III malocclusion, facial profile, and molar relationship reported in our study [10].

Reported prevalence of increased overjet ranges between 11.5% [6] and 28.1% [12] which is noticeably lower than our results (36.1%). In our study, a reduced overjet was also higher than previous reported figures (for Jordanians (8.6%) [10], Saudians (11.4%) [13], and Nigerians (8.3%) [16]). Some of these Indifferences may be due to different study thresholods/criteria to categorize excessive overjet.

our study, the overbite was recorded as deep if the upper incisors covered ˃one third of the lower incisor clinical crown length, with prevalence which is higher than previously reported figures and almost double that reported for Jordanian school children (16.9%) [10]. There is, however, marked differences in thresholds for this assessment between studies [11, 12, 14, 15, 18].

Judgment of AOB is less variable, but prevalence of AOB of was again double that reported for Jordanian school children (2.9%) [10] although within a previously reported rang (1.6% [12] to 15% [6]).

#### DHC and IOTN

In the present study the number of subjects in severe and extreme need of orthodontic treatment (grades 4 and 5).was double the reported results of a Saudi study (21%) [13]. Other studies report definite need for orthodontic treatment ranging from 28–40% [14, 34, 35]. In our study, if the borderline-need cases are also considered, 67.6% of the refugee population are in need of orthodontic treatment.

The main limitation of the present study was the lack of radiographic diagnostic aids. The dental units in Zaatari camp lacked any radiographic equipment and were equipped only for emergency treatments. Additional, it was not deemed ethical to carryout full radiographic examination merely for epidemiological data, especially given that any such examination (unfortunately) would not be followed by any needed orthodontic treatment, due to limited provision and lack of funding: it is anticpated that the epidemiological data from this study would support and help to get the fund needed to treat these refugees. Despite this limitation, this study is the first to fill the gap in literature by reporting on the malocclusion patterns of children/adolescent Syrian refugees and their orthodontic needs, highlighting some of the challenges that are faced by this underprivileged population. Moreover, the data provide a preliminary reference for dental practitioners, researchers and policy makers to develop and implement community-based services and preventive and interceptive programs for this population.

## Conclusions

There is a high prevalence of malocclusion in Syrian refugee school children/adolescents with more than two thirds in moderate to severe need of orthodontic treatment, higher than other international host populations. This data highlights an urgent need to provide targeted dental services and to decide treatment priorities among those with moderate to severe orthodontic treatment needs.

## Data Availability

All collected data from patients analyzed during this study are included in this published article. The datasets used and analyzed during the current study are available from the corresponding author on reasonable request.

## Abbreviations

AOB: Anterior open bite
DHC: Dental health component
FMPA: Frankfort-mandibular plane angle
IOTN: Index of orthodontic treatment need
NLA: Nasolabial angle
OB: Overbite
OJ: Overjet
RCP–ICP: Retrusive contact position–intercuspal position
